# Age prediction using fundus parameters of normal eyes from the Kumejima population study

**DOI:** 10.1007/s00417-024-06471-4

**Published:** 2024-05-31

**Authors:** Takehiro Yamashita, Hiroto Terasaki, Ryo Asaoka, Aiko Iwase, Hiroshi Sakai, Taiji Sakamoto, Makoto Araie

**Affiliations:** 1 Department of Ophthalmology, Kagoshima University Graduate School of Medical and Dental Sciences, Kagoshima, Japan; 2 Department of Ophthalmology, Seirei Hamamatsu General Hospital, Shizuoka, Japan; 3 Tajimi Iwase Eye Clinic, Gifu, Japan; 4 Urasoe Sakai Eye Clinic, Okinawa, Japan; 5 Department of Ophthalmology, Kanto Central Hospital, Tokyo, Japan

**Keywords:** Color fundus photographs, Aging, Population study, Fundus parameters, Age

## Abstract

**Purpose:**

Artificial intelligence can predict the age of an individual using color fundus photographs (CFPs). This study aimed to investigate the accuracy of age prediction in the Kumejima study using fundus parameters and to clarify age-related changes in the fundus.

**Methods:**

We used nonmydriatic CFPs obtained from the Kumejima population study, including 1,646 right eyes of healthy participants with reliable fundus parameter measurements. The tessellation fundus index was calculated as R/(R + G + B) using the mean value of the red–green–blue intensity in eight locations around the optic disc and foveal region. The optic disc ovality ratio, papillomacular angle, and retinal vessel angle were quantified as previously described. Least absolute shrinkage and selection operator regression with leave-one-out cross-validation was used to predict age. The relationship between the actual and predicted ages was investigated using Pearson’s correlation coefficient.

**Results:**

The mean age of included participants (834 males and 812 females) was 53.4 ± 10.1 years. The mean predicted age based on fundus parameters was 53.4 ± 8.9 years, with a mean absolute error of 3.64 years, and the correlation coefficient between actual and predicted age was 0.88 (*p* < 0.001). Older patients had greater red and green intensities and weaker blue intensities in the peripapillary area (*p* < 0.001).

**Conclusions:**

Age could be predicted using the CFP parameters, and there were notable age-related changes in the peripapillary color intensity. The age-related changes in the fundus may aid the understanding of the mechanism of fundus diseases such as age-related macular degeneration.


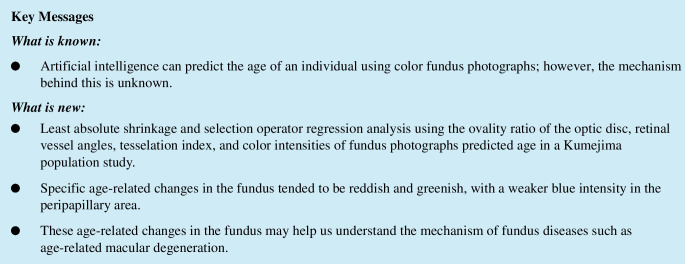


## Introduction

The ocular fundus, like the face, varies from person to person, and the variation is great enough to be used for personal identification [1]. This implies that the fundus contains several pieces of information that can identify an individual. Artificial intelligence (AI), particularly deep learning AI, can estimate age, sex, systolic blood pressure, smoking habits, presence of cardiac complications [2], refraction [3], and axial length [4] using only fundus photographs. In particular, age can be estimated with a mean absolute error (MAE) within 3.26 years [2], which is surprising to ophthalmologists. However, it is impossible to identify which factors are important for estimating the age of individuals, even when using attention heat maps or quantifying a few factors. Similar problems occur with chess, Go, and Shogi, in which deep-learning AI can predict the best move or the result; however, it is unknown why the predicted move is superior [5], and professional chess players need to study the reasons for AI moves. Thus, it is difficult for professionals to clarify the reason for selections made by deep learning AI; this is also called black box AI [6]. Furthermore, the fundus is more complicated than what is in these games because the eyes are natural objects.

Conventional statistical methods such as multiple regression analysis using many factors have been proposed as possible explanatory AI to unravel this black box [6]. Like the face, the fundus is unique and has many different features. For example, there are large individual variations in the angle or trajectory of the retinal vessels [7–11], the location and shape of the optic disc [12, 13], and the color of the peripapillary area [14–17]. The authors have previously reported that regression with L2 regularization (ridge regression) using these fundus parameters could determine sex with an accuracy rate of 77.9% in young adults (persons in their 20 s) [18], 63.2% in 8.5-year-olds [19], and 80.4% in a study population (> 40 years old) [20]. Sex-specific differences were also found because multiple regression analysis was used. The optic disc was oval-shaped, the retinal vessels were closer to the fovea, and the peripapillary color of the fundus was greenish in females than in males [18–20].

Therefore, the authors hypothesized that this method could predict age from fundus parameters and reveal specific age-related changes in the fundus. Thus, this study aimed to predict the age of older individuals using the regression analyses of their color fundus photographs (CFPs). The CFPs of 1,646 eyes from individuals ≥ 40 years old who were participants in the Kumejima population study were investigated to accomplish this.

## Methods

### Study population

The study adhered to the principles outlined in the Declaration of Helsinki and followed Japanese regulations. The protocol received approval from the Ethics Board of the Regional Council, and written informed consent was obtained from all participants prior to their involvement.

Kumejima island spans an area of 63.2 square kilometers and is situated in the southwestern region of Japan. It lies west of the main island of Okinawa and has a population of around 9000, primarily consisting of individuals from Okinawa Prefecture. The study was conducted from May 2005 to August 2006, during which all residents aged 40 and above were informed about the study’s protocol and invited to participate. In 2005, the official household registration database indicated that Kumejima had 5249 residents aged 40 and above. After excluding 617 residents for various reasons, a total of 4632 individuals were eligible for the study [21, 22].

### Examination and diagnosis

The screening process involved a structured interview, the specifics of which were previously documented in a published paper [21, 22]. Ocular examinations were conducted by skilled ophthalmologists and examiners. These examinations included assessments of best-corrected visual acuity, spherical equivalent (ARK-730, Topcon, Japan), intraocular pressure measured with a Goldmann applanation tonometer, and axial length (IOLMaster, Carl Zeiss Meditec, Germany). Additionally, slit-lamp biomicroscopy, gonioscopy, ophthalmoscopy, fundus photography, and perimetry were performed. Sequential stereoscopic color fundus photographs (CFPs) with a 45-degree angle were obtained using a nonmydriatic digital ocular fundus camera system (Image Net TRC-NW7, Topcon, Japan). Detailed analyses of the optic disc, fundus, visual field examinations, and diagnosis of ocular diseases have been extensively described elsewhere [21, 22].

### Measurement of fundus parameters

**Fig. 1:**
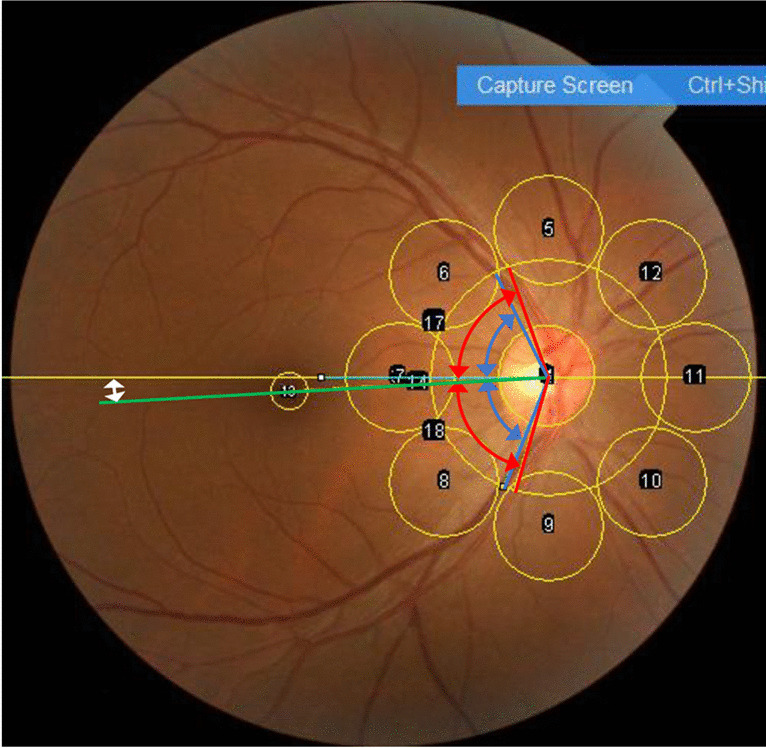
Method of quantifying retinal vessel angles and papillomacular position (PMP), ovality ratio, and red–green–blue intensity. Red double arrows indicate supratemporal and infratemporal retinal artery angles (ST-RA and IT-RA). Blue double arrows indicate the supratemporal and infratemporal retinal vein angles (ST-RV, IT-RV). The white double arrow indicates PMP. The ovality ratio was determined by dividing the maximum diameter by the minimum diameter of the optic disc. The red-green-blue intensities and the tesselation fundus index (TFI) were calculated for each of the eight locations around the optic disc and fovea

Following the method of our prior investigation, we measured 42 fundus parameters [20] (Fig. 1). The specifics of these measurements have been previously documented. We determined the angles of the supratemporal (ST) and infratemporal (IT) major retinal arteries (RA), as well as the angles of the retinal veins (VA) against the temporal horizontal line. Additionally, we measured the ST-RA, IT-RA, ST-VA, and IT-VA angles [7, 8, 11]. The papillomacular position (PMP) was defined as the angle formed by a horizontal line intersecting with a line connecting the optic disc center to the fovea [12].

We calculated the ovality ratio by dividing the maximum disc diameter by the minimum disc diameter [13]. Furthermore, we computed the mean red, green, and blue intensities within each area for the eight foveal and peripapillary circles. The tessellation fundus index (TFI) was determined using the mean red (R), green (G), and blue (B) intensities at each of the nine locations with the formula: TFI = R/(R + G + B) [14, 15].

These measurements were conducted utilizing color fundus photography (CFP) images and the ImageJ software (version 1.47, National Institutes of Health, Bethesda, MD, USA; available at http://imagej.nih.gov/ij/). The macro function of ImageJ facilitated semi-automated calculation of these 42 CFP parameters once the locations of the fovea, optic nerve head edge, and crossing points of the supralateral and inferolateral retinal arteries or veins were determined.

### Statistical analyses

As in our previous studies, least absolute shrinkage and selection operator (LASSO) regression [23, 24] with 42 variables, RGB intensities, and TFI in nine locations (ST-RA, IT-RA, ST-VA, IT-VA, PMP, and ovality ratio) was used to predict age [25, 26]. LASSO regression with leave-one-out cross-validation was used to overcome the overfitting problem in ordinal statistical regression models, such as multivariate linear regression or binomial logistic regression, by applying a penalty to coefficients (L1 regularization), and the sum of the absolute values of the regression coefficients was regularized [23–26]. More precisely, let x ∈ R^p denote the variables and let y ∈ R denote the response (please note x_ij are normalized and y has mean zero). The Lasso algorithm solves the following problem:

1 $$\underset{\left(\beta 0, \beta \right)\epsilon {R}^{p+1}}{{\text{min}}}[\frac{1}{2N}\sum_{i=1}^{N}(yi- \beta 0-{x}_{i}^{Y}\beta {)}^{2}+ \lambda {P}_{\alpha }(\beta )],$$

2 $${P}_{\alpha }\left(\beta \right)=\left(1-\alpha \right)\frac{1}{2} \left|\left|\beta \right|{|}_{{l}_{2}}^{2}+ \alpha \right|\left|\beta \right|{|}_{{l}_{1}}$$

3 $$=\sum\nolimits_{j=1}^{p}[\frac{1}{2} (1-\mathrm{\alpha }){\beta }_{j}^{2}+ \alpha |{\beta }_{j}|]$$

In the Eq. (1), $\underset{\left(\beta 0, \beta \right)\epsilon {R}^{p+1}}{{\text{min}}}[\frac{1}{2N}\sum_{i=1}^{N}(yi- \beta 0-{x}_{i}^{Y}\beta {)}^{2}]$ is identical to OLSLR and $\lambda {P}_{\alpha }(\beta )$ is the penalty term for the shrinkage [23, 24].

The advantage of this approach lies in its ability to directly observe the effects of parameters in the optimal model, akin to ordinal multiple linear/logistic regression, unlike deep learning. The diagnostic performance of the LASSO binomial logistic regression approach was assessed using the leave-one-out cross-validation method. Here, each eye from the original dataset served as validation data iteratively, while the remaining observations (1645 eyes) were designated as training data [20, 27]. This process was repeated for each eye, resulting in 1646 iterations. Diagnostic accuracy was evaluated using the area under the receiver operating characteristic curve (ROC AUC). The final optimal model was determined using all 1646 eyes.

Pearson’s correlation analysis was employed to ascertain the relationship between actual and predicted ages, as well as the relationships between age and fundus parameters. Given the relatively large study sample, the significance level was set at *p* < 0.001. All statistical analyses were conducted using SPSS Statistics 19 for Windows (SPSS Inc., IBM, Somers, New York, USA) and R (version 3.1.3; R Foundation for Statistical Computing, Vienna, Austria).

## Results

Color fundus photographs (CFPs) of acceptable quality were obtained from 3762 participants, totaling 7524 eyes. However, photographs from 376 right eyes and 421 left eyes were excluded due to conditions such as cataract, corneal opacity, large pterygium, or a small pupil. Additionally, eyes with a history of intraocular surgery (453 right, 443 left) were excluded. Eyes with a refractive error exceeding − 8 or + 5 diopters (14 right eyes, 11 left eyes), or those with optic disc diseases including glaucoma or retinal/brain diseases in either eye (711 right eyes, 679 left eyes) were also excluded.

Ultimately, both eyes of 2208 individuals were deemed suitable for analysis. Further details regarding the demographics of these eyes were provided in a previous report. [20, 28, 29] Only the right eyes were utilized for measuring fundus parameters. Among the 2208 normal eyes, 562 were excluded due to difficulties in measuring fundus parameters arising from unclear peripheral fundus areas or unidentifiable foveal regions. Consequently, 1646 right eyes were included in the analyses. Table 1 presents demographic data for the right eyes of eligible participants. All participants were over 40 years old and exhibited no significant myopia.

**Table 1:** Participant data

Male 834 eyes, Female 812 eyes	Mean ± standard deviation (range)
Age (years)	53.4 ± 10.1 (40–80)
Refractive error (diopters)	-0.14 ± 1.62 (-7.625–5.500)
Axial length (mm)	23.50 ± 0.88 (20.92–27.55)

**Fig. 2:**
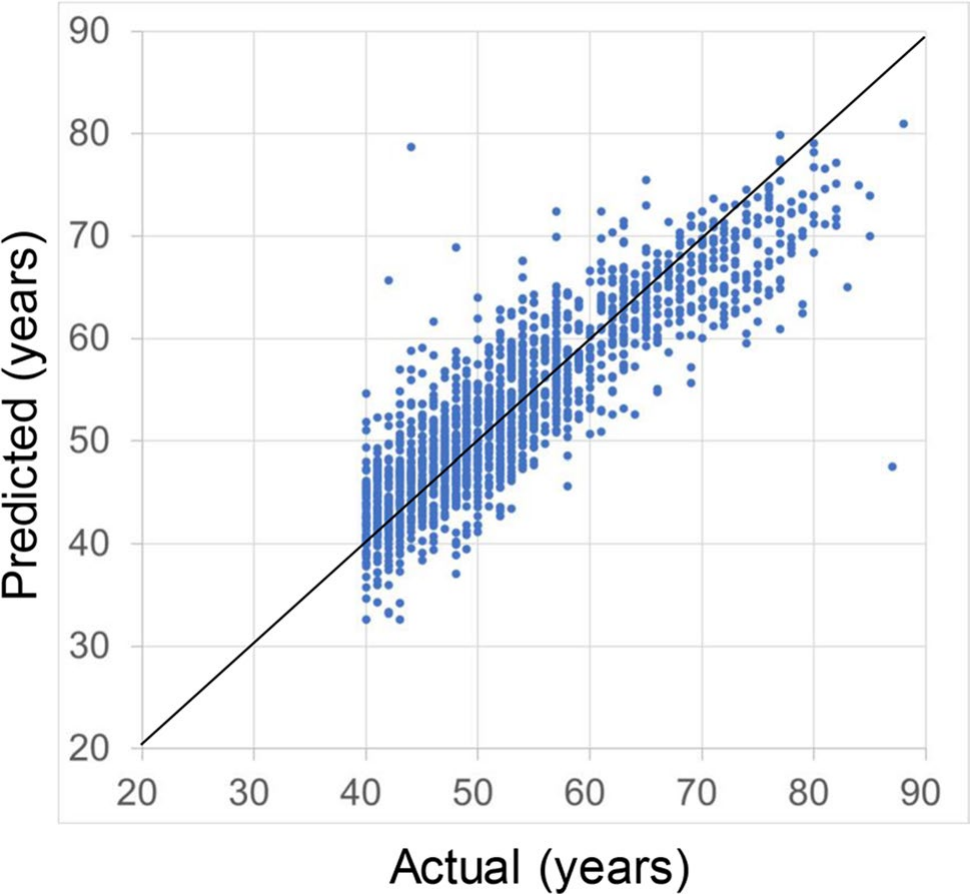
Scatter plot analysis between actual and predicted age. The line represents actual = predicted values

The MAE for predicting the age of participants was 3.64 years (95% confidence interval: 3.48–3.80) (Table 2). The correlation coefficient between the actual and predicted age was 0.88 (*p* < 0.001) (Fig. 2). The optimal model for age obtained using LASSO regression and Pearson’s correlation coefficient between the actual age and fundus parameters is shown in Table 3. Older eyes tended to have (i) larger ST-RA; (ii) higher red intensity in all areas; (iii) higher green intensity except in the supratemporal, temporal, and infratemporal areas; (iv) lower blue intensity except in the infranasal, nasal, supranasal, superior, and fovea areas; and (v) higher TFI except in the supranasal and nasal areas.

**Table 2:** Prediction accuracy of this and previous studies

	This study (Regression analysis)	UK biobank [2] (Artificial intelligence)	EyePACS-2 K [2] (Artificial intelligence)
Actual age (years)	53.4 ± 10.1	56.9 ± 8.2	54.9 ± 10.9
Predicted age (years)	53.4 ± 8.9	Not available	Not available
Mean absolute error (years)	3.64	3.26	3.42
Correlation coefficient	0.88	0.86	0.91

**Table 3:** Optimal model for the age obtained using the LASSO binomial logistic regression and Pearson’s correlation between actual age and fundus parameters

Fundus parameters		Coefficient of LASSO regression	Pearson’s correlation Coefficient	*p*-value of Pearson’s correlation
Papillomacular position (PMP)		-0.0657	0.076	0.002
Ovality ratio		-1.1813	0.012	0.631
Retinal artery angle	Supratemporal (ST-RA)	0.0033	0.086	< 0.001
	Infratemporal (IT-RA)	-0.0049	0.044	0.075
Retinal vein angle	Supratemporal (ST-RV)	0.0141	0.058	0.018
	Infratemporal (IT-RV)	0.0073	0.059	0.017
Red intensity (R)	Temporal	-0.0001	0.155	< 0.001
	Supratemporal	N.S	0.195	< 0.001
	Superior	0.0033	0.266	< 0.001
	Supranasal	-0.0595	0.206	< 0.001
	Nasal	-0.0740	0.136	< 0.001
	Infranasal	0.0137	0.298	< 0.001
	Inferior	0.0168	0.267	< 0.001
	Infratemporal	-0.0086	0.158	< 0.001
	Fovea	0.2668	0.253	< 0.001
Green intensity (G)	Temporal	0.1568	-0.051	0.040
	Supratemporal	-0.1051	0.064	0.009
	Superior	0.0186	0.260	< 0.001
	Supranasal	0.2031	0.255	< 0.001
	Nasal	0.0391	0.203	< 0.001
	Infranasal	0.2156	0.333	< 0.001
	Inferior	N.S	0.154	< 0.001
	Infratemporal	-0.0343	-0.064	0.009
	Fovea	-0.4701	0.464	< 0.001
Blue intensity (B)	Temporal	-0.6893	-0.438	< 0.001
	Supratemporal	0.2172	-0.295	< 0.001
	Superior	0.1620	-0.052	0.034
	Supranasal	-0.2274	0.003	0.891
	Nasal	-0.0723	0.032	0.197
	Infranasal	0.0246	0.046	0.061
	Inferior	-0.0651	-0.223	< 0.001
	Infratemporal	-0.0404	-0.442	< 0.001
	Fovea	0.2321	0.458	< 0.001
Tessellation fundus index (TFI)	Temporal	N.S	0.457	< 0.001
	Supratemporal	N.S	0.376	< 0.001
	Superior	34.9370	0.160	< 0.001
	Supranasal	-0.1719	0.053	0.030
	Nasal	-10.7043	-0.009	0.727
	Infranasal	-17.9720	0.097	< 0.001
	Inferior	N.S	0.351	< 0.001
	Infratemporal	55.3161	0.482	< 0.001
	Fovea	-126.5101	-0.489	< 0.001

N.S.: not selected, ST-RA: supra temporal retinal artery angle, IT-RA: infra temporal retinal artery angle, ST-RV: supra temporal retinal vein angle, IT-RV: infra temporal retinal vein angle

**Fig. 3:**
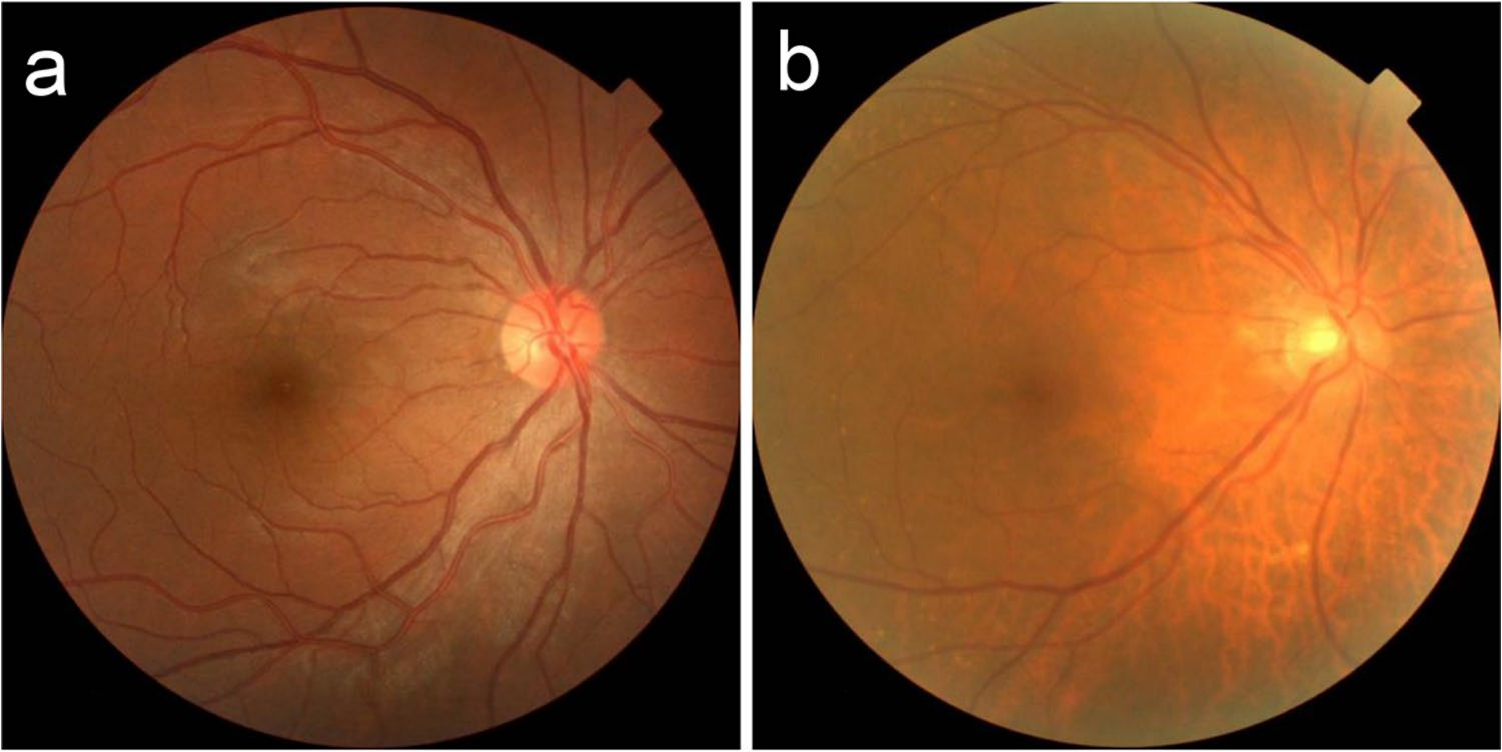
Typical color fundus images of younger and older eyes. **a** a younger eye (actual age: 40 years old, predicted age: 44 years old, axial length: 23.17 mm) and (**b**) an older eye (actual age: 80 years old, predicted age: 78 years old, axial length: 23.27 mm)

The typical color fundus images of (**a**) a younger eye (actual age: 40 years, predicted age: 44 years, axial length: 23.17 mm) and (**b**) an older eye (actual age: 80 years, predicted age: 78 years, axial length: 23.27 mm) are shown in Fig. 3.

## Discussion

The accuracy of age prediction using LASSO regression in the Kumejima population study was similar to that in a previous report of deep learning AI, also a cross-sectional study, with participants > 40 years old. In general, the prediction accuracy of deep learning AI outperformed that of the regression analysis. For example, in sex determination, deep learning AI had a 97% accuracy rate compared to 80.4% for regression analysis [2, 20]. One reason regression analysis was as accurate as AI for age prediction was that the participants in the AI study included eyes with fundus diseases such as diabetic retinopathy. Thus, the pathological findings may have masked age-related changes. Another possibility is that the prediction may have been highly accurate because no other fundus photographic parameters significantly affected age-related changes. This can be explained by the parameters measured in this study, especially fundus color intensity.

Pearson’s correlation coefficient showed that the red intensity tended to become stronger in older age groups. The retina [30] and retinal pigment epithelial cells [31] become thinner with age, suggesting that the increased visibility of the choroid is responsible for the red intensity. Additionally, tessellation tends to be stronger in older individuals. It has been reported that the choroid [32] becomes thinner with age, and the thinner the choroid, the stronger the tessellation [33]. These findings are consistent with the results of the present study.

The green intensity tended to become stronger in older age except in the supratemporal, temporal, and infratemporal areas. A typical fundus photograph of an older person shows a greenish color in the periphery (Fig. 3b). This greenish appearance of the periphery may be due to the presence of cataracts. Since all cases with poor fundus photographs were excluded, there were no cases of strong cataracts. However, since the lens becomes cloudy with age, the effects of cataracts are inevitable. If this is the case, it is not a true fundus change; nonetheless, because cataracts tend to become stronger with age, it could be said that some age-related changes are visible in fundus photographs. However, no published evidence supports this hypothesis. Hence, further research is needed to determine what happens to the greenish areas in the periphery of fundus photographs before and after cataract surgery.

The blue intensity tended to be weaker in older age groups, except in the superior, supranasal, nasal, and infranasal areas. Conversely, the blue intensity at the posterior pole tended to be stronger in younger patients. Blue light is mainly reflected from the retinal surface, and the retinal light reflex weakens with age [34]. These results may have been caused by decreased retinal surface reflex.

Since the angle of ST-RA is wider in older persons, the retinal vessel angle is unlikely to widen with age. The Kumejima Study was a 2005 cross-sectional study; the older the patient, the shorter the axial length and the more hyperopic the refraction [28]. The correlation coefficient between age and axial length in this study was -0.219 (*p* < 0.001), and that between age and refractive error (spherical equivalent) was 0.472 (*p* < 0.001). The arterial vessel angle tends to be larger, with shorter axial length and more hyperopic refraction [7]. Since this was a cross-sectional AI study [2], the vessel changes may be generational differences (cohort effect) rather than the aging effect. Further studies using fundus photographs from long-term cohorts are needed to determine true age-related changes.

Red, green, and blue colors tended to become stronger in the fovea of older individuals. Red and green areas tended to increase with age, as indicated by the peripapillary area; however, the opposite trend was observed for blue areas. The cause of this trend toward a blueish fovea in older participants is unknown. The TFI at the fovea tended to be weaker in older patients. Although foveal TFI has not been reported, more extensive macular TFI tends to be stronger in older patients. The retinal pigment epithelial layer in the macula thins with age [35], but that in the fovea thickens with age [36]. These results may explain the opposite trends in TFI between the macula and fovea.

Because the fundus parameters interacted with each other, the results of the LASSO regression differed in part from Pearson’s correlation results. Blue and green intensities may be more important than red intensities and TFI in estimating age because both blue and green showed similar trends, but red and TFI showed opposite trends for some factors. Our previous reports on sex determination using ridge regression analysis [20] and attention heap map analysis in an AI study ^2^ revealed that comprehensive judgment using many factors could achieve high accuracy. It is difficult for humans to make comprehensive judgments using many factors; however, multiple regression analysis can help identify important factors related to age-related changes in the fundus.

This study had some limitations. First, this was a cross-sectional study, similar to previously reported AI studies. Hence, future longitudinal cohort studies are needed to investigate the true age-related changes in the fundus. Second, this study was an epidemiological study of persons aged ≥ 40 years and thus did not include young adults aged 20–40 years. Therefore, the results for young adults are unknown. In addition, because the study was conducted in Japan, the results may differ in other racial groups. Finally, the exclusion criteria employed reduced the number of participants and thus the applicability of the results. However, the exclusion criteria were deemed necessary to ensure the quality of the fundus photographs to allow for the measurement of various parameters.

In conclusion, LASSO regression analyses using parameters from CFPs can predict age in the Kumejima population to the same extent as deep learning AI. Specific age-related changes in the fundus tended to be reddish and greenish, with weaker blue intensity in the peripapillary area. These age-related changes in the fundus may aid the understanding of the mechanism of fundus diseases such as age-related macular degeneration.

## References

[1] Lawrence S, Giles CL, Tsoi AC, Back AD (1997) Face recognition: a convolutional neural-network approach. IEEE Trans Neural Netw 8(1):98–113. 10.1109/72.554195 18255614 10.1109/72.554195

[2] Poplin R, Varadarajan AV, Blumer K et al (2018) Prediction of cardiovascular risk factors from retinal fundus photographs via deep learning. Nat Biomed Eng 2(3):158–164. 10.1038/s41551-018-0195-0 31015713 10.1038/s41551-018-0195-0

[3] Varadarajan AV, Poplin R, Blumer K et al (2018) Deep learning for predicting refractive error from retinal fundus images. Invest Ophthalmol Vis Sci 59(7):2861–2868. 10.1167/iovs.18-23887 30025129 10.1167/iovs.18-23887

[4] Dong L, Hu XY, Yan YN et al (2021) Deep learning-based estimation of axial length and subfoveal choroidal thickness from color fundus photographs. Front Cell Dev Biol 9:653692. 10.3389/fcell.2021.653692 33898450 10.3389/fcell.2021.653692 PMC8063031

[5] David OE, van den Herik HJ, Koppel M, Netanyahu NS (2013) Genetic algorithms for evolving computer chess programs. IEEE Trans Evol Computat 18(5):779–789. 10.1109/TEVC.2013.2285111

[6] Bathaee Y (2018) The artificial intelligence black box and the failure of intent and causation. Harv JL & Tech 31:889–938

[7] Yamashita T, Asaoka R, Tanaka M et al (2013) Relationship between position of peak retinal nerve fiber layer thickness and retinal arteries on sectoral retinal nerve fiber layer thickness. Invest Ophthalmol Vis Sci 54(8):5481–5488. 10.1167/iovs.12-11008 23847316 10.1167/iovs.12-11008

[8] Yamashita T, Asaoka R, Kii Y, Terasaki H, Murata H, Sakamoto T (2017) Structural parameters associated with location of peaks of peripapillary retinal nerve fiber layer thickness in young healthy eyes. PLoS ONE 12(5):e0177247. 10.1371/journal.pone.0177247 28542289 10.1371/journal.pone.0177247 PMC5444611

[9] Yamashita T, Sakamoto T, Terasaki H, Tanaka M, Kii Y, Nakao K (2014) Quantification of retinal nerve fiber and retinal artery trajectories using second-order polynomial equation and its association with axial length. Invest Ophthalmol Vis Sci 55(8):5176–5182. 10.1167/iovs.14-14105 25074777 10.1167/iovs.14-14105

[10] Yamashita T, Terasaki H, Yoshihara N, Kii Y, Uchino E, Sakamoto T (2018) Relationship between retinal artery trajectory and axial length in Japanese school students. Jpn J Ophthalmol 62(3):315–320. 10.1007/s10384-018-0572-y 29442204 10.1007/s10384-018-0572-y

[11] Fujino Y, Yamashita T, Murata H, Asaoka R (2016) Adjusting circumpapillary retinal nerve fiber layer profile using retinal artery position improves the structure-function relationship in glaucoma. Invest Ophthalmol Vis Sci 57(7):3152–3158. 10.1167/iovs.16-19461 27309619 10.1167/iovs.16-19461

[12] Garway-Heath DF, Poinoosawmy D, Fitzke FW, Hitchings RA (2000) Mapping the visual field to the optic disc in normal tension glaucoma eyes. Ophthalmology 107(10):1809–1815. 10.1016/s0161-6420(00)00284-0 11013178 10.1016/s0161-6420(00)00284-0

[13] Tay E, Seah SK, Chan SP et al (2005) Optic disk ovality as an index of tilt and its relationship to myopia and perimetry. Am J Ophthalmol 139(2):247–252. 10.1016/j.ajo.2004.08.076 15733984 10.1016/j.ajo.2004.08.076

[14] Yoshihara N, Yamashita T, Ohno-Matsui K, Sakamoto T (2014) Objective analyses of tessellated fundi and significant correlation between degree of tessellation and choroidal thickness in healthy eyes. PLoS ONE 9(7):e103586. 10.1371/journal.pone.0103586 25068821 10.1371/journal.pone.0103586 PMC4113439

[15] Yamashita T, Terasaki H, Tanaka M, Nakao K, Sakamoto T (2020) Relationship between peripapillary choroidal thickness and degree of tessellation in young healthy eyes. Graefes Arch Clin Exp Ophthalmol 258(8):1779–1785. 10.1007/s00417-020-04644-5 32248408 10.1007/s00417-020-04644-5

[16] Yan YN, Wang YX, Xu L, Xu J, Wei WB, Jonas JB (2015) Fundus tessellation: prevalence and associated factors: the Beijing Eye Study 2011. Ophthalmology 122(9):1873–1880. 10.1016/j.ophtha.2015.05.031 26119000 10.1016/j.ophtha.2015.05.031

[17] Yamashita T, Iwase A, Kii Y et al (2018) Location of ocular tessellations in Japanese: population-based Kumejima study. Invest Ophthalmol Vis Sci 59(12):4963–4967. 10.1167/iovs.18-25007 30326064 10.1167/iovs.18-25007

[18] Yamashita T, Asaoka R, Terasaki H et al (2020) Factors in color fundus photographs that can be used by humans to determine sex of individuals. Transl Vis Sci Technol 9(2):4. 10.1167/tvst.9.2.4 32518709 10.1167/tvst.9.2.4 PMC7255626

[19] Noma S, Yamashita T, Asaoka R et al (2020) Sex judgment using color fundus parameters in elementary school students. Graefes Arch Clin Exp Ophthalmol 258(12):2781–2789. 10.1007/s00417-020-04969-1 33064194 10.1007/s00417-020-04969-1

[20] Yamashita T, Asaoka R, Iwase A et al (2023) Sex determination using color fundus parameters in older adults of Kumejima population study. Graefes Arch Clin Exp Ophthalmol 261(8):2411–2419. 10.1007/s00417-023-06024-1 36856844 10.1007/s00417-023-06024-1

[21] Sawaguchi S, Sakai H, Iwase A et al (2012) Prevalence of primary angle closure and primary angle-closure glaucoma in a southwestern rural population of Japan: the Kumejima Study. Ophthalmology 119(6):1134–1142. 10.1016/j.ophtha.2011.12.038 22361313 10.1016/j.ophtha.2011.12.038

[22] Yamamoto S, Sawaguchi S, Iwase A et al (2014) Primary open-angle glaucoma in a population associated with high prevalence of primary angle-closure glaucoma: the Kumejima Study. Ophthalmology 121(8):1558–1565. 10.1016/j.ophtha.2014.03.003 24746386 10.1016/j.ophtha.2014.03.003

[23] Tibshirani R (1996) Regression shrinkage and selection via the lasso. J R Stat Soc B 58(1):267–288. 10.1111/j.2517-6161.1996.tb02080.x

[24] Friedman J, Hastie T, Tibshirani R (2010) Regularization paths for generalized linear models via coordinate descent. J Stat Softw 33(1):1–22. 10.1109/TPAMI.2005.127 20808728 PMC2929880

[25] Asaoka R (2013) Measuring visual field progression in the central 10 degrees using additional information from central 24 degrees visual fields and “lasso regression.” PLoS ONE 8(8):e72199. 10.1371/journal.pone.0072199 23951295 10.1371/journal.pone.0072199 PMC3741185

[26] Fujino Y, Murata H, Mayama C, Asaoka R (2015) Applying, “lasso” regression to predict future visual field progression in glaucoma patients. Invest Ophthalmol Vis Sci 56(4):2334–2339. 10.1167/iovs.15-16445 25698708 10.1167/iovs.15-16445

[27] Japkowicz N (2011) Evaluating learning algorithms: a classification perspective. Cambridge University Press

[28] Yamashita T, Iwase A, Sakai H, Terasaki H, Sakamoto T, Araie M (2019) Differences of body height, axial length, and refractive error at different ages in Kumejima study. Graefes Arch Clin Exp Ophthalmol 257(2):371–378. 10.1007/s00417-018-4192-5 30506096 10.1007/s00417-018-4192-5

[29] Iwase A, Sawaguchi S, Sakai H, Tanaka K, Tsutsumi T, Araie M (2017) Optic disc, rim and peripapillary chorioretinal atrophy in normal Japanese eyes: the Kumejima Study. Jpn J Ophthalmol 61(3):223–229. 10.1007/s10384-017-0499-8 28185066 10.1007/s10384-017-0499-8

[30] Poon LY, Antar H, Tsikata E et al (2018) Effects of age, race, and ethnicity on the optic nerve and peripapillary region using spectral-domain OCT 3D volume scans. Transl Vis Sci Technol 7(6):12. 10.1167/tvst.7.6.12 30510856 10.1167/tvst.7.6.12 PMC6262887

[31] Bhatia SK, Rashid A, Chrenek MA et al (2016) Analysis of RPE morphometry in human eyes. Mol Vis 22:898–916 27555739 PMC4968610

[32] Jiang R, Wang YX, Wei WB, Xu L, Jonas JB (2015) Peripapillary choroidal thickness in adult Chinese: the Beijing Eye study. Invest Ophthalmol Vis Sci 56(6):4045–4052. 10.1167/iovs.15-16521 26114482 10.1167/iovs.15-16521

[33] Yamashita T, Sakamoto T, Yoshihara N et al (2017) Correlations between local peripapillary choroidal thickness and axial length, optic disc tilt, and papillo-macular position in young healthy eyes. PLoS ONE 12(10):e0186453. 10.1371/journal.pone.0186453 29023585 10.1371/journal.pone.0186453 PMC5638527

[34] Dieck S, Ibarra M, Moghul I et al (2020) Factors in color fundus photographs that can be used by humans to determine sex of individuals. Transl Vis Sci Technol 9(7):8. 10.1167/tvst.9.7.8 32832215 10.1167/tvst.9.7.8 PMC7414790

[35] Ghanem Kadhim Z, Mohammad NK (2023) Effect of aging and lifestyle on healthy macular photoreceptors and retinal pigment epithelium-Bruch membrane complex thickness. Eur J Ophthalmol 33(1):441–447. 10.1177/11206721221101372 35585693 10.1177/11206721221101372

[36] Harris J, Subhi Y, Sørensen TL (2017) Effect of aging and lifestyle on photoreceptors and retinal pigment epithelium: cross-sectional study in a healthy Danish population. Pathobiol Aging Age Relat Dis 7(1):1398016. 10.1080/20010001.2017.1398016 29152163 10.1080/20010001.2017.1398016 PMC5678353

